# Moving proteins along in the cilium

**DOI:** 10.7554/eLife.55254

**Published:** 2020-02-13

**Authors:** Narcis Adrian Petriman, Esben Lorentzen

**Affiliations:** Department of Molecular Biology and GeneticsAarhus UniversityAarhusDenmark

**Keywords:** BBSome, cryo-em, *bos taurus*, arl6, cilia, intraflagellar transport, Human, Other

## Abstract

The structures of the bovine and human BBSome reveal that a conformational change is required to recruit the complex to the ciliary membrane.

**Related research article** Singh SK, Gui M, Koh F, Yip MCJ, Brown A. 2020. Structure and activation mechanism of the BBSome membrane protein trafficking complex. *eLife* **9**:e53322. doi: 10.7554/eLife.53322

**Related research article** Klink BU, Gatsogiannis C, Hofnagel O, Wittinghofer A, Raunser S. 2020. Structure of the human BBSome core complex. *eLife* **9**:e53910. doi: 10.7554/eLife.53910

Bardet-Biedl syndrome is a genetic disease that causes blindness, obesity and kidney anomalies (Brown and Witman, 2014; Sheffield, 2010). It arises due to mutations in more than 20 genes, eight of which encode the proteins that form a large complex known as the BBSome (Nachury et al., 2007). This complex is found in the cilia that protrude from most eukaryotic cells and have important roles in motility and signaling (Wingfield et al., 2018). It is in charge of exporting membrane proteins from the cilium to the cell body through a process known as intraflagellar transport (Lechtreck et al., 2009; Figure 1).

**Figure 1.**
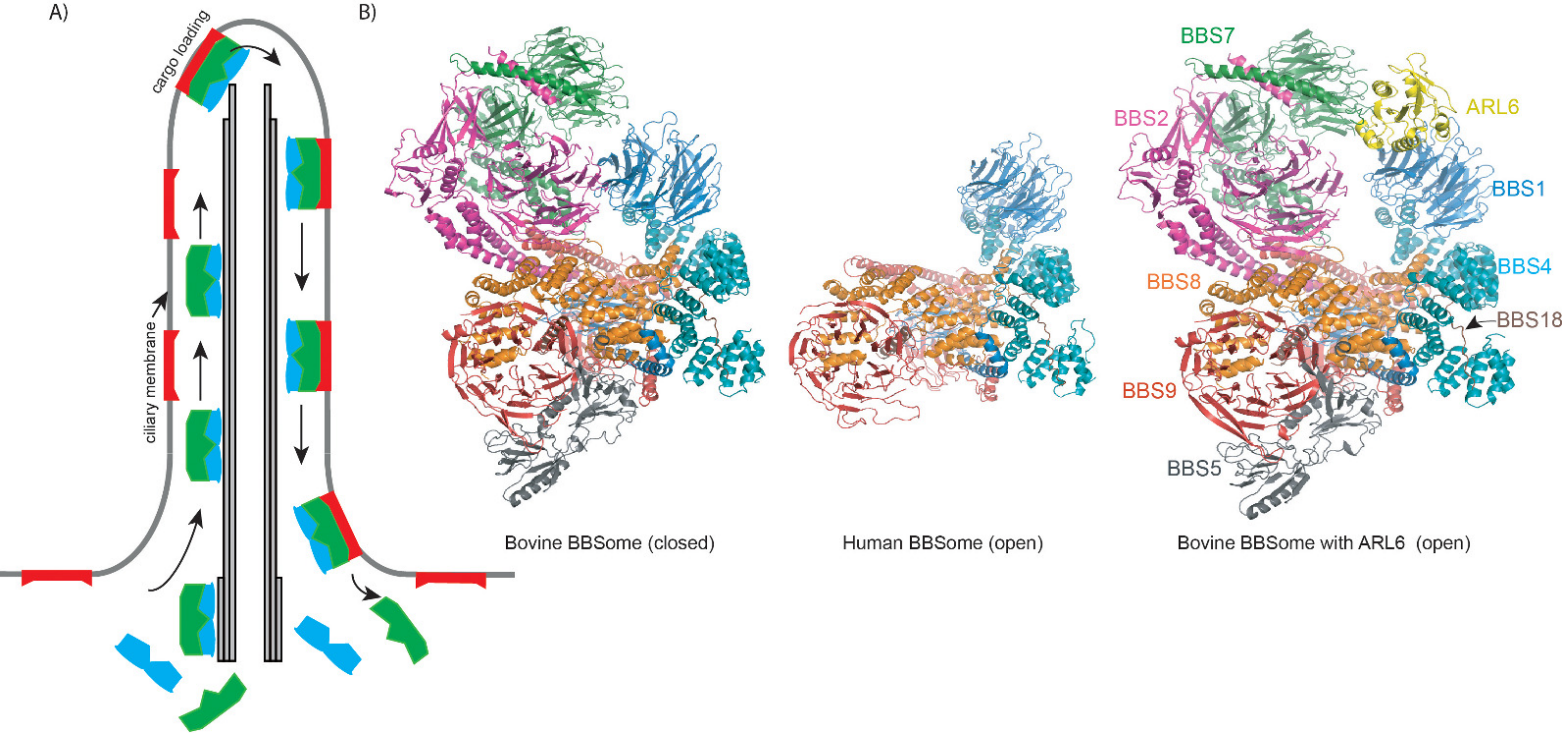
Export of ciliary signaling factors by the BBSome. (**A**) Highly simplified schematic of the intraflagellar transport cycle in a cilium. The closed BBSome complex (green) binds the intraflagellar transport machinery (IFT, blue) at the base of the cilium and starts travelling towards the tip of the cilium along a structure called the axoneme (grey), which is formed by microtubules. Once near the tip, the complex binds ARL6, becoming activated, and it can take ciliary membrane proteins (red) as cargo and transport them to the base of the cilium. At this point, the BBSome releases its cargo (which remains embedded in the membrane) and dissociates from the IFT machinery. (**B**) Cartoon representation of closed and open conformations of bovine and human BBSome complexes with each subunit represented in a different color.

The details of how the BBSome proteins organize into a complex that can recognize signaling molecules in the cilium membrane for intraflagellar transport are not fully understood. Now, in eLife, two independent teams report how they have purified BBSome complexes and used cryo-electron microscopy to determine their structure with a resolution that reveals new insights into how they work. In the first study Alan Brown and colleagues at Harvard Medical School – including Sandeep Singh as first author – report the results of experiments on BBSomes from the bovine retina (Singh et al., 2020). In the second study, Stefan Raunser and colleagues at the Max Planck Institute of Molecular Physiology – including Björn Klink as first author – report the structure of the human BBSome (Klink et al., 2020).

The high resolution of the newly reported structures allowed the accurate visualization of amino acid side-chains in the complex, something that was not possible in a medium-resolution structure that had been published previously (Chou et al., 2019). This high resolution also enabled Singh et al. to map out the mutations reported in patients with Bardet-Biedl syndrome onto the three-dimensional structure of the BBSome, revealing that they cluster in specific regions.

The overall architecture of the human and bovine BBSomes is very similar and immediately reveals the protein interactions involved in the formation of the complex (Figure 1). Many of these interactions occur between protein domains known as β-propeller, tetratricopeptide, coil-coiled and γ-adaptin ear domains. The complex is glued together by BBS18, a mostly unstructured protein that snakes its way through the BBSome and interacts with several of the other proteins in the complex (Figure 1). Interestingly, the complex adopts both an open and a closed conformation.

Why does the BBSome adopt two different conformations? It has to be near the ciliary membrane to transport signaling factors embedded in the membrane. The complex becomes activated when it binds to ARL6, a small GTPase associated to the membrane, but to bind this protein the BBSome must be in an open conformation (Jin et al., 2010). However, the medium-resolution structure of the mouse BBSome reported previously revealed a closed conformation that was not compatible with another published structure for the BBS1-ARL6 subcomplex (Chou et al., 2019; Mourão et al., 2014). This suggests that the complex must undergo a large conformational change to accommodate ARL6 and facilitate membrane recruitment.

Singh et al. report structures for the BBSome on its own and for the complex binding ARL6, and they propose a mechanism for how it is activated. This mechanism involves the BBS1 β-propeller swiveling into a new position and the β-sheet between the BBS1 and the BBS2 subunits being broken. Many of the reported mutations in patients with Bardet-Biedl syndrome are in the regions that change conformation when ARL6 binds, suggesting the mutations may interfere with BBSome activation.

The conformational change in the proposed mechanism opens up the BBSome, which likely exposes the binding site for the proteins that the complex transports. This site may recognize specific amino acid sequences in certain ciliary signaling proteins, explaining why the BBSome is selective in the proteins it transports. The way the BBSome exposes its binding site may be similar to the mechanism adopted by certain vesicle-coating complexes, and many of the proteins in the BBSome are structurally similar to the proteins in these complexes (Jin et al., 2010; Singh et al., 2020).

The human BBSome structure reported by Klink et al. is also in the open conformation. The complex has a prominent negatively-charged patch on its surface, which is likely involved in the recognition of positively-charged regions in the proteins it traffics to and from the ciliary membrane.

With the BBSome structure revealed, several open questions remain. How does the complex specifically recognize sequences in the ciliary signaling factors it transports? And how does it couple to the intraflagellar transport machinery? The work of Singh et al. and Klink et al. lays out the basis for future work to analyze the BBSome in complex with various signaling factors. This work will likely reveal why the BBSome only transports a subset of ciliary signaling factors.
